# Mangiferin (mango) attenuates AOM-induced colorectal cancer in rat’s colon by augmentation of apoptotic proteins and antioxidant mechanisms

**DOI:** 10.1038/s41598-023-50947-y

**Published:** 2024-01-08

**Authors:** Khaled Abdul-Aziz Ahmed, Ahmed A. J. Jabbar, Mahmood Ameen Abdulla, Zaenah Zuhair Alamri, Nur Ain Salehen, Ibrahim Abdel Aziz Ibrahim, Ghassan Almaimani, Ghazi A. Bamagous, Riyad A. Almaimani, Hussain A. Almasmoum, Mazen M. Ghaith, Wesam F. Farrash

**Affiliations:** 1 Department of Medical Laboratory Sciences, Faculty of Allied Medical Sciences, Al-Ahliyya Amman University, Amman, Jordan; 2 Department of Medical Laboratory Technology, Erbil Technical Health and Medical College, Erbil Polytechnic University, Erbil, 44001 Iraq; 3 Department of Medical Microbiology, College of Science, Cihan University-Erbil, Erbil, Kurdistan Region Iraq; 4 Department of Biological Science, College of Science, University of Jeddah, P.O. Box 80327, Jeddah, 21589 Saudi Arabia; 5 Department of Biomedical Sciences, Faculty of Medicine, University of Malaya, 50603 Kuala Lumpur, Malaysia; 6 Department of Pharmacology and Toxicology, Faculty of Medicine, Umm Al-Qura University, Makkah, Saudi Arabia; 7 Department of Surgery, Faculty of Medicine, Umm Al-Qura University, Al Abdeyah, PO Box 7607, Makkah, Saudi Arabia; 8 Department of Biochemistry, Faculty of Medicine, Umm Al-Qura University, Makkah, Saudi Arabia; 9 Department of Clinical Laboratory Science, Faculty of Applied Medical Sciences, Umm Al-Qura University, Makkah, Saudi Arabia

**Keywords:** Biochemistry, Immunology, Physiology, Gastroenterology, Medical research

## Abstract

Mangiferin (MF) is a natural C-glucosylxantone compound that has many substantial curative potentials against numerous illnesses including cancers. The present study's goal is to appraise the chemo preventive possessions of MF on azoxymethane (AOM)-mediated colonic aberrant crypt foci (ACF) in rats. Rats clustered into 5 groups, negative control (A), inoculated subcutaneously with normal saline twice and nourished on 0.5% CMC; groups B-E injected twice with 15 mg/kg azoxymethane followed by ingestion of 0.5% CMC (B, cancer control); intraperitoneal inoculation of 35 mg/kg 5-fluorouracil (C, reference rats) or nourished on 30 mg/kg (D) and 60 mg/kg (E) of MF. Results of gross morphology of colorectal specimens showed significantly lower total colonic ACF incidence in MF-treated rats than that of cancer controls. The colon tissue examination of cancer control rats showed increased ACF availability with bizarrely elongated nuclei, stratified cells, and higher depletion of the submucosal glands compared to MF-treated rats. Mangiferin treatment caused increased regulation of pro-apoptotic (increased Bax) proteins and reduced the β-catenin) proteins expression. Moreover, rats fed on MF had significantly higher glutathione peroxidase (GPx), superoxide dismutase (SOD), catalase (CAT), and lower malondialdehyde (MDA) concentrations in their colonic tissue homogenates. Mangiferin supplementation significantly down-shifted pro-inflammatory cytokines (transforming growth factor-α and interleukine-6) and up-shifted anti-inflammatory cytokines (interleukine-10) based on serum analysis. The chemo-protective mechanistic of MF against AOM-induced ACF, shown by lower ACF values and colon tissue penetration, could be correlated with its positive modulation of apoptotic cascade, antioxidant enzymes, and inflammatory cytokines originating from AOM oxidative stress insults.

## Introduction

Colorectal cancer (CRC) is a deleterious malignant neoplasm recognized as the third dominant leading risk factor of death-associated cancer in all nations. And it's the second leading cause of mortality-related cancer when statistics were combined from both genders^1,2^. The risk factor for acquiring colorectal cancer is slightly higher in females (1–26) than in males (1–23) during their lifetime. However, this range changes based on colorectal risk factors such as stress, alcohol, malnutrition, smoking, and obesity^3^. Despite pharmaceutical revolutionary innovations for designing effective anti-cancer drugs against colorectal cancer, all of these synthetic chemicals come with many drawbacks in the short and long terms including hair loss, neuropathy, nephropathy, digestive problems, and sexual disability^4^. Therefore, searching for alternative natural medicine (chemoprotective) with lower side effects is crucial for lowering the mortality and morbidity rate related to colorectal cancer. Alternatively, plants and their active ingredients serve as excellent chemotherapy without any noticeable adverse effects. For example, mangiferin displayed inhibitory efficacy against numerous cancer cells including HeLa S3 and KBvin cells^5^, acute leukemia cells^6^, and colon cancer^7^. The colon carcinogenesis model (induced by AOM) is the most commonly used method to assess the chemoprotective role of a particular ingredient in an animal model^8^. Colorectal cancer incidence in rats is based on different factors including dosage, frequency, duration of ingestion, and route of ingestion^9^. Moreover, other factors such as gender, age, and genomic format of animals could also play a role in the initiation and development of colorectal cancer^10^.

A plethora of folkloric herbal medicine utilized as cancer therapeutics and cancer prevention without any adverse effects^8,11–14^. Phytochemicals namely alkaloids are among the most common chemicals heavenly investigated in the aspect of chemoprotection^15^ and multidrug resistance^16^. Flavonoids as secondary metabolites have shown numerous biological activities, antimicrobial^17^, antivirus^18^, anti-inflammatory^19^, anticancer^20^, and antimutagenic actions^21^. An example of such a natural product is mangiferin, a natural glucoxilxanthone compound obtained primitively and chiefly from plant species *Mangifera indica* (Mango) and *Salicia chinesis* (saptarangi)^22^. Mounting Pharmacological evidence has indicated numerous bioactivity and therapeutic actions of mangiferin. More recently, scientists have shown different biological potentials of mangiferin compounds including antioxidant^23^, antimicrobial^24^, antifungal^25^, anti-diabetic^26^, analgesia^27^, anti-inflammatory^28^, anti-apoptotic actions^29^, and cardioprotective^30^.

Mangiferin is a common xanthone (1,3,6,7-tetrahydroxyxanthone C2-β-d-glucoside) bioactive compound that are detected in different plant species belonging to Anacardiaceae and Gentianaceae families including higher plants (*Iris unguicularis)* and honeybush. The structural alignment of xanthonoids in this compound plays a key role in the recognition and binding of mangiferin with specific receptors of synthetic drugs. Mangiferin has shown similar drug characteristics, weight of particles, catechol moiety, and estimated partition coefficient (C Log P) number, which facilitates its pharmacological potential. Studies revealed mangiferin potentials in conjugate with phospholipids that will enhance intestinal permeability^31^ and can combine with β-cyclodextrin causing up-surging of water and thermal stability^32^. Moreover, mangiferin has a stable linkage between its C-glucosyl and aromatic-hydroxyl groups correlated majorly with its antioxidant and iron chelation potentials^33^. Accordingly, the antiradical potentials of mangiferin were found in various organs (heart, liver, kidney, and lungs)^34^. Thus, recent outcomes present mangiferin as a valuable bioactive natural chemical for alternative medicine against an extensive range of oxidative stress-related health issues.

Inflammation is a series of pathological processes that can be initiated as a result of prolonged oxidative stress including stimulation of the NF-κB mechanism, increased expression of cyclooxygenase-2 (COX-2), production of NO by stimulatory nitric oxide synthase (iNOS)^35^. Moreover, inflammation may also result from the pathogenic entrance that leads to up-regulation of the pro-inflammatory cytokine, tumor necrosis factor-alpha (TNF-α), interleukin-6, IL-8, chemokines CCL2, and CXCL8, while reducing anti-inflammatory cytokines (IL-10)^8,14^. Chronic inflammation (CI), a long-term health defect has been correlated with many lifelong series diseases namely, atherosclerosis, cardiovascular diseases (CVD), inflammatory bowel disease (IBD), kidney disease, and diabetes mellitus. CI is labelled as one of the main leading causes of death worldwide according recent estimation, which revealed that half of all deaths across nations result from inflammation-related diseases and autoimmune diseases^36^. IBD is an inflammatory-mediated gastrointestinal disease recognized by disruption of intestinal epithelium (intestinal barrier), which usually prevents penetrations of pathogens and toxic compounds and permits passage of only certain micromolecules (nutrients and electrolytes) through different ion and protein channels. Chronic inflammation can disrupt the characteristic selective permeability of the intestinal defence layer causing the passage of macromolecules (pathogens, exotoxins, and fats) from the lumen into the intestinal tissues as commonly known as leaky gut, consequently, this will lead to colorectal cancer. Therefore, controlling inflammation is a crucial step toward the prevention of colorectal cancer especially in IBD patients^37^. Despite numerous investigations (in vitro and in vivo) on the pharmacological potentials of MF, however, it’s in vivo, colon cancer cytotoxicity and its underline mechanism are yet to be found.

Herein, we rationally designed the current experiment to evaluate the chemoprotective potentials of MF in AOM-induced oxidative stress-mediated colorectal cancer in rats. Here we studied the in vivo gross morphology, colonic histopathology, immunohistochemistry, antioxidant enzymatic and non-enzymatic, inflammatory cytokines, and blood biochemical parameters upon AOM-induced colorectal cancer in the presence of different dosages of MF.

## Materials and methods

### Ethic approval for the animal experiment

The study protocols was carried out in compliance with the ARRIVE guidelines and in accordance with guidelines set by Iraqi animal rights and National scientific recommendations for laboratory animal experiments^38^. The current animal procedure was agreed upon by the Ethics Committee of Cihan University-Erbil (BIO/11/12/2022/M.A.A.).

### Acute toxicity

The toxicity of the MF was assessed to ensure its safety on experimental rats. Fifteen rats were randomly segregated into the three cages, group 1 (normal control), received 10% Tween 20; Group 2, administered a low dosage of MF; Group 3, rats ingested a high dosage of MF based on the OECD guideline^39^. Rats had no access of food prior 24 hours of the supplementation. Treated rats (G2 and G3) were administered single dose of 250 and 500 mg/kg of MF by oral gavage, respectively. Food was removed for another 3 to 4 h after MF ingestion and the record begin immediately after treatment and continued for 14 days (every 8 h) for any possible toxic or physiological changes. The observational process continued for detecting any abnormalities, or death during and after 14 days of the experiment. Directly after two weeks, rats received an overdose of anesthesia [xylazine (12.5 mg/kg) and ketamine (87.5 mg/kg)] and sacrificed. Intracardial blood samples centrifuged (centrifuge, LC carousel, Roche, Germany) and serum separated for biochemical analysis^40^. The liver and kidney organs examined of any histological changes as a result of MF ingestion^41^.

### Chemoprotection procedure of MF

#### Experimental design

Thirty adult Dawley rats (male) were arbitrarily aligned into 5 cages (6 rats in each). A, normal control rats; B, cancer control rats; C, Reference rats received 5-FU (5-fluorouracil); D and E, rats were supplemented with a low and high dose of MF^8^.

**Figure 1:**
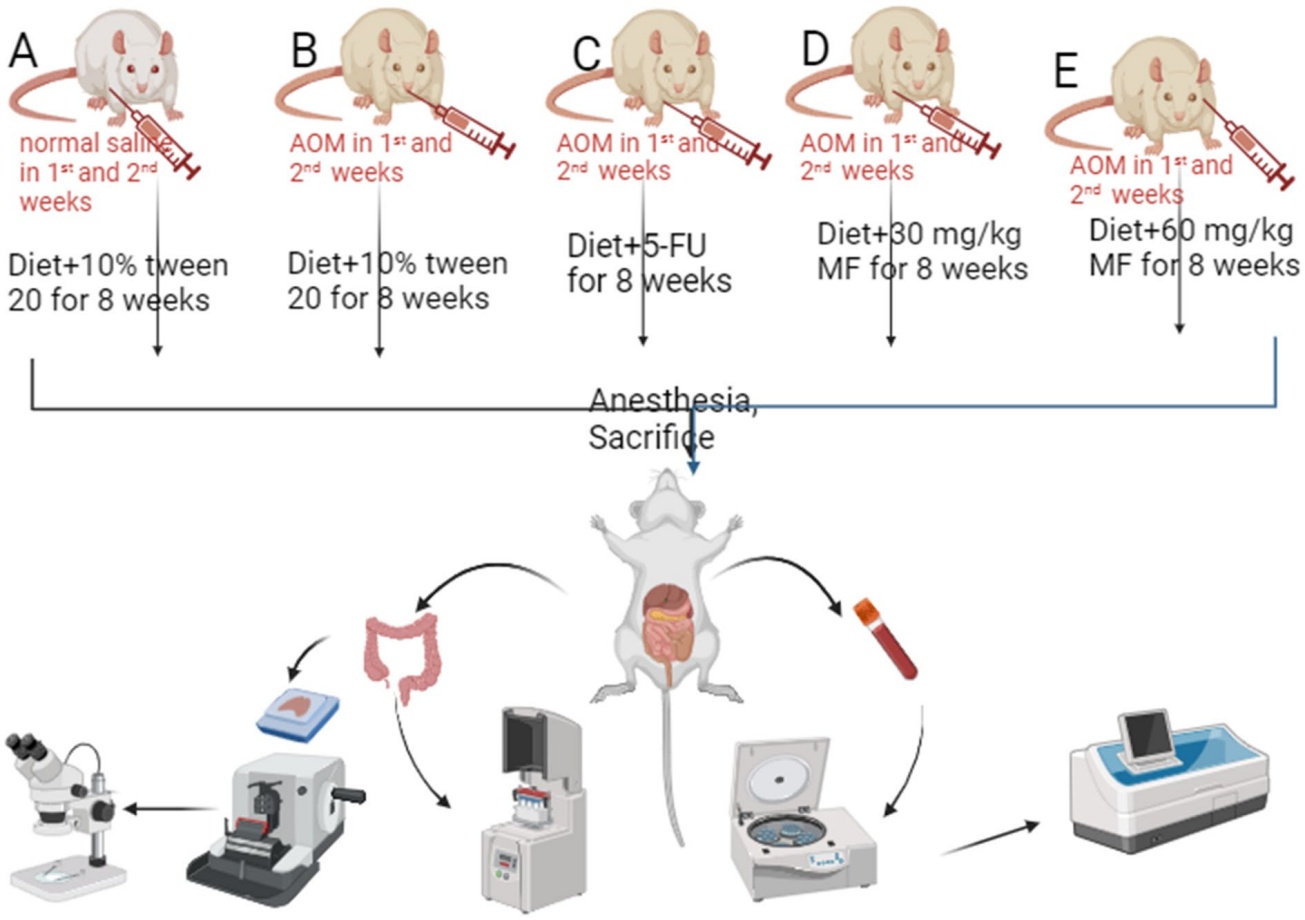
Schematic timeline of experimental design. Created in Biorender (by A.A.J.). (**A**) Normal control rats; (**B**) cancer control rats; (**C**) reference rats; rats received 5-FU (5-fluorouracil); (**D**, **E**) rats were supplemented with 30 and 60 mg/kg MF.

Normal control rats received a saline solution of 15 mg/kg and rats in group B-E were given two doses of 15 mg/kg AOM twice in two weeks by subcutaneous injection. In addition, normal and cancer control rats were given 15 mg/kg of 10% Tween 20 (5 mL/kg); reference rat control had 5-FU (5-fluorouracil) by intravenous injection, and MF-treated rats received 30, and 60 mg/kg by oral gavage for two months. After that, rats received overdose of anaesthesia [xylazine (12.5 mg/kg) and ketamine (87.5 mg/kg)] and sacrificed (Fig. 1A–E). The colon specimens were collected from all experimental rats and examined for the degree of produced ACF by different histopathology techniques. Tissue samples were transferred into liquid nitrogen to undergo homogenization process^42^.

#### Evaluation of ACF scores

The experimental rats were given anaesthesia, sacrificed, and the colon tissues were mixed with cold phosphate-buffered saline (PBS). Longitudinal cutting of colon tissues was made from the bottom to the rectum. After that, tissues were coloured with methylene blue dye (0.2%) for the microscopic examination and measurement of ACF degrees. The ACF scores were determined for each tissue specimen by estimation of ACF in different microscopic focus^43^.

$$ {\text{Inhibition (}}\% ) \, = {\text{ Total no}}.{\text{ of foci in negative control}}/{\text{no}}.{\text{ of foci in each group }} \times { 1}00. $$

#### Histology procedure of ACFs

Colon tissue samples were mixed with Buffered formalin (10%) as the preparation technique for the machinery tissue process (Leica, Germany). After that, tissues were blocked with paraffin, and a regular slice of 5-mm was set on slides and coloured with hematoxylin and eosin (H&E). The histological examination of stained slides was made using a light microscope (Nikon, Japan)^11^.

#### Immunohistochemistry

Briefly, colon tissues have undergone a process of de-paraffinization and rehydration, and mixing with 10 mM sodium citrate buffer (10 min) for antigen retrieval. The temperature of tissue samples was cooled down by Tris-buffered saline before the antioxidant procedure using an ARK peroxidase kit (DAKO Denmark A/S, Glostrup, Denmark). Tissue mixing with peroxidase solution enables the blockage of endogenous hydrogen peroxidase 0.5% (5 min). Finally, colon tissues were dehydrated and prepared on slides for the incubation procedure (20 min) by biotinylated antibodies versus Bax and β-catenin (Elabscience, USA) and then, followed by the addition of the streptavidin–HRP (nutrient) and deepen in 3-3- diaminobenzidine as chromogen for 10 min. After washing, the slide transferred into Mayer's haematoxylin, dried, and mounted for microscopic examination^12^.

#### Antiradical evaluation of homogenized colon

The colons were put in ice-cold saline for the homogenization procedure, by using ice-cold phosphate buffer (10% w/v, 50 mM, pH 7.4), mammalian protease inhibitor, and centrifuge (30 min at 10,000 *g* at 4 °C). The supernatant was moved into separate tubes and investigated for the antioxidant enzymes (CAT, SOD, GPX) and MDA content (kits from Elabscience, USA)^14^.

#### Measurement provocative cytokines

The obtained serum specimen analysed for TNF-α, IL-6, and IL-10 content by using an ELISA kit My BioSource, USA. The procedure followed the standards set by producer company. Cytokine strength was found by normal sanitized recombinant cytokines^14^.

#### Biochemical analysis

The current study evaluated the liver synthetic function by estimation of total protein and Albumin levels in plasma samples along with liver enzymes (AST, ALT, and GGT). Kidney functionality was assessed by evaluation of urea creatinine concentrations in plasma specimens obtained from different experimental groups. Blood samples from all experimental rats were taken to laboratory for evaluation of different liver and kidney parameters by an automatic analyser, Cobas c311 (Roche, USA) using Cobas commercial rat kits^44^.

### Statistics

Data results are seen as mean ± SEM resulting from triplicate analysis. The statistical method for the current study was one-way analysis (ANOVA, SPSS software) and Graph Pad prism 9.0. The current significant value was set at *P*< 0.05.

## Results

### Acute toxicity

**Figure 2:**
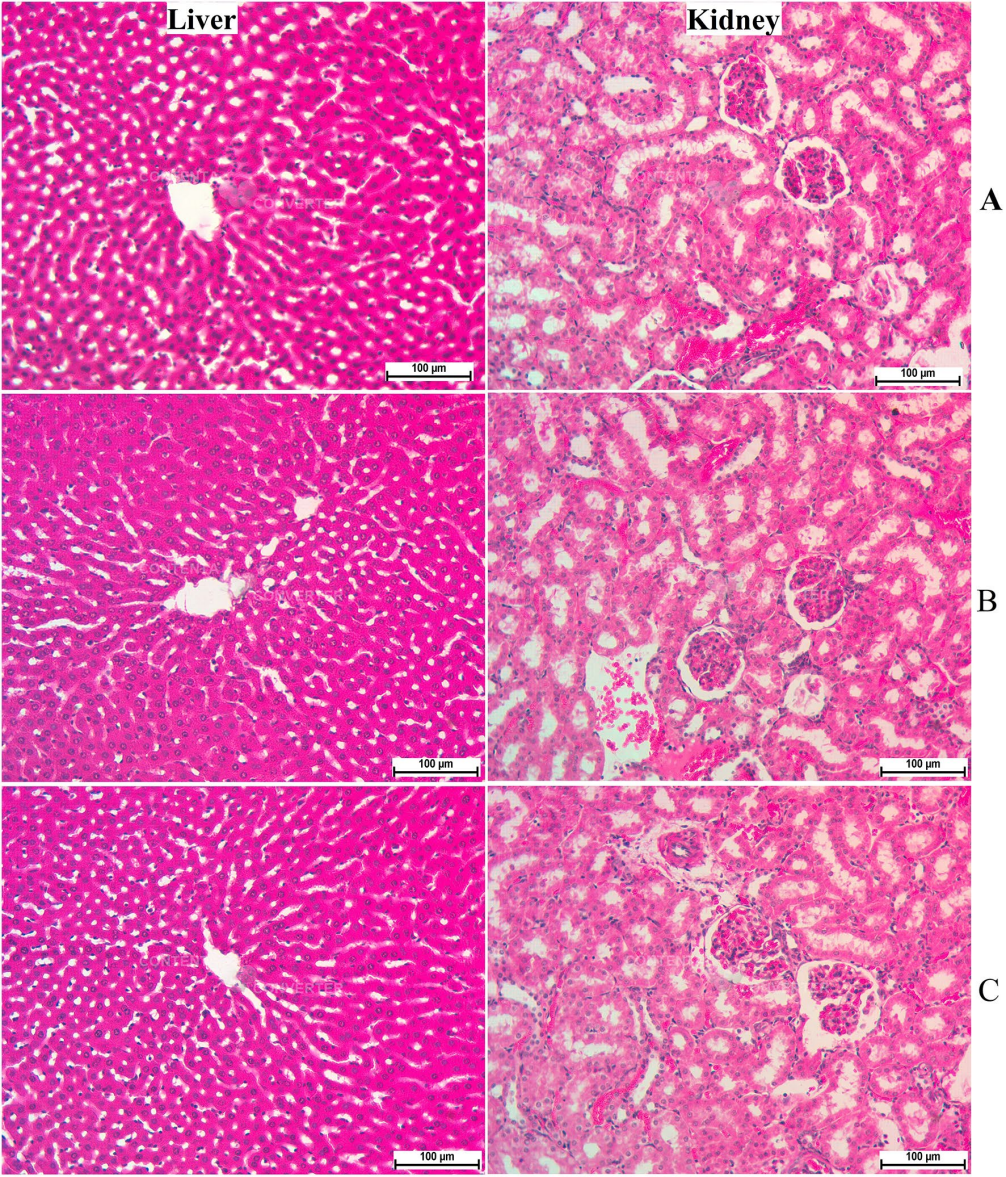
Microscopic views of renal and hepatic tissues in acute toxicity test. (**A**) Normal control rats received 10% Tween20; (**B**) and (**C**) rats received 250 and 500 mg/kg of MF by oral gavage, respectively (magnification, ×20).

The current trial showed the safety of MF supplementation in 250 and 500 mg/kg doses for 14 days. Continuous observation (every 8 h) did not show any abnormal features in the physiology or appearance of rats. Moreover, physical activity and feed intake were very comparable between MF-ingested rats and normal control rats. Histological examination showed comparable tissue structure of kidney and liver tissues obtained from normal control and MF-treated rats (Fig. 2). The current results suggest the toxicity of MF may occur at doses higher than 500 mg/kg MF as rats were completely healthy even after the experiment.

### In vivo anticancer effects of MF

#### Number of foci in colon

**Figure 3:**
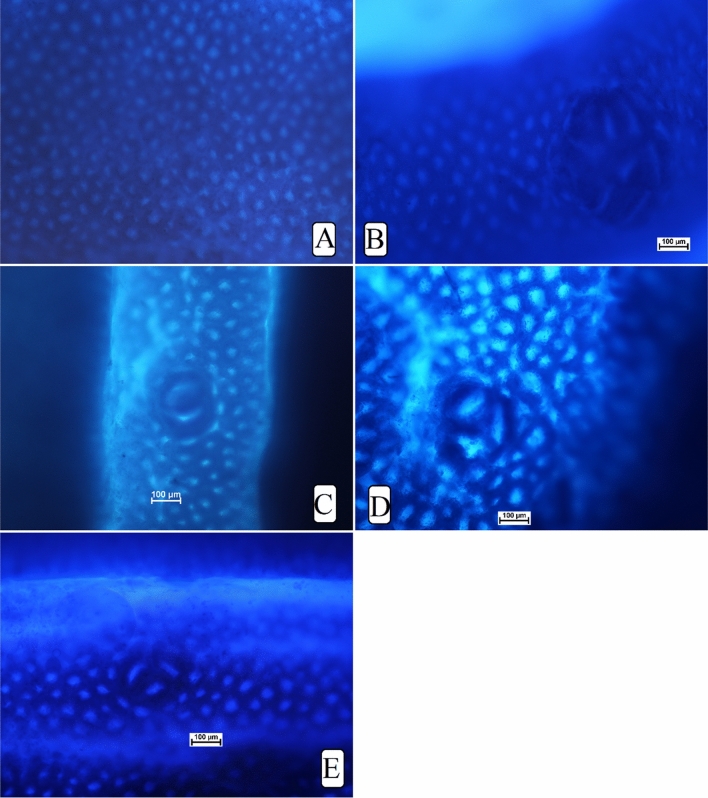
Gross morphology of ACF in colon tissues of rats. (**A**) Normal negative rats; (**B**) cancer rats treated only with AOM; (**C**) reference rats received 35 mg/kg of 5-FU; (**D**, **E**) rats received 30 and 60 mg/kg MF (magnification, ×10).

The aberrant crypt foci were observed in all rats injected with AOM (Table 1 and Fig. 3). incidence in the colon (proximal and distal parts) was significantly higher in positive control rats (B) compared to rats treated with 5-FU (C) or MF (30 and 60 mg/kg) as shown in Table 1. However, increased ACF values were detected in the distal colon, regardless of different rat ingestions. Rats ingested 60 mg/kg (E) had significantly lower foci numbers (in the proximal and distal colon) than that of positive controls but the values were not statistically different compared to that of 5-FU-treated rats. The inhibition percentage of foci was significantly reduced by MF treatment. Rats fed on a diet supplemented with 60 mg/kg MF showed significantly lower total ACF (36.95) and inhibition percentage (60.78%) than that (94.22 and 0%) of cancer controls.

**Table 1:** Effects on the ACF values in experimental rats.

Groups	Crypt 1	Crypt 2	Crypt 3	Crypt ≥ 4	Total ACF	Inhibition %
A	N/A	N/A	N/A	N/A	N/A	N/A
B	8.3 ± 1.4^b^	22.32 ± 3.9^c^	28.8 ± 2.4^c^	34.8 ± 2.1^c^	94.22^a^	0
C	3.5 ± 1.3^a^	6.16 ± 0.75^a^	6.12 ± 0.7^a^	15.16 ± 1.7^a^	30.94^c^	67.16^a^
D	6.66 ± 0.4^a^	10.33 ± 1.02^b^	11.2 ± 1.2^b^	25 ± 2.6^b^	53.19^b^	43.54^b^
E	3.65 ± .9^a^	9 ± 1.2^b^	7.5 ± 1.0^a^	16.8 ± 0.7^a^	36.95^c^	60.78^a^

Numbers are available as means ± SD (n = 12). Means with shared letters indicate non-significant at (p < 0.05). (A) Normal negative rats; (B) cancer rats treated only with AOM, (C) reference rats received 35 mg/kg of 5-FU; (D, E) rats received 30 and 60 mg/kg MF.

The outcomes indicate a significant difference in the ACF formation between normal and treated rats. Rats had only AOM (B) showed numerous foci in both of their colon parts with many ACF aggregations compared to other treated rat groups. Mangiferin (60 mg/kg) treatments lead significant reduction in the ACF values in different areas of their colon. Gross morphology of colon tissues by using methylen blue elucidate different level of the colonic tissue damages in different (Fig. 3).

#### Histopathology of AOM-induced foci in colon

**Figure 4:**
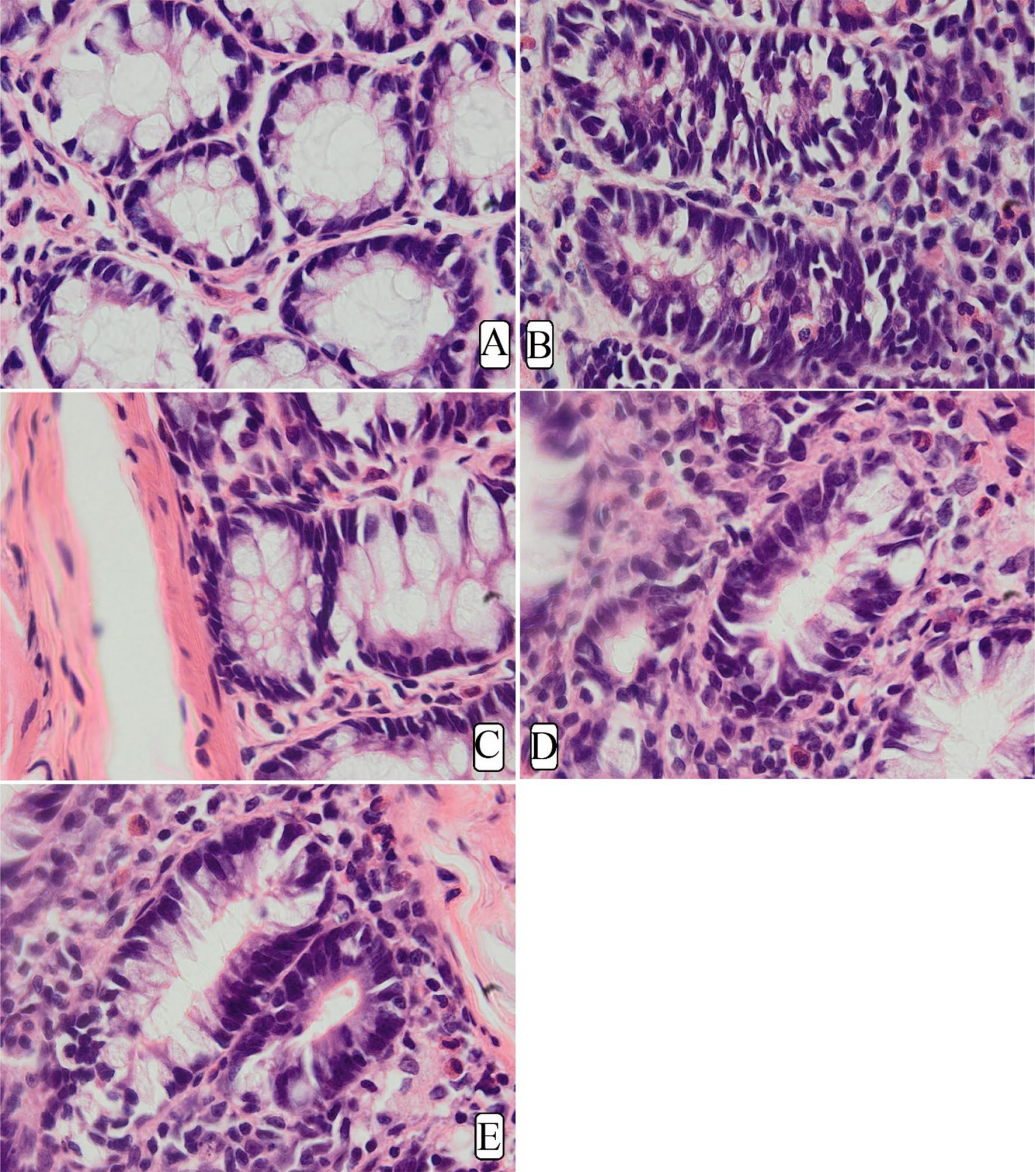
Microscopic views of cross-sectioned colonic tissues in rats. (**A**) Normal negative rats; (**B**) cancer rats treated only with AOM; (**C**) reference rats received 35 mg/kg of 5-FU; (**D**, **E**) rats received 30 and 60 mg/kg MF. Hematoxylin and eosin (H&E) (magnification, ×100). Rats showed a degree of submucosal penetrations, nucleus malformation, and interstitial inflammations.

The results have shown that AOM induction caused significant colon tissue injury represented by glandular dysplasia in the submucosal layer featured with inflammatory cells (Fig. 4). The glandular dysplasia was distorted in many rows or grouped near the lumen. Rats experienced foci (Fig. 4B–E) and showed numerous epithelial cells with dense mucin, pleomorphic nucleus, reduced cell polarity, mitotic hyperactivity (hyperchromasia), anisocytosis, and absence of goblet cells. Rats treated with MF had lower colon damage with atypical epithelial cells, normal mucin thickness, less nucleus malformation, and normal mitotic action (Fig. 4). Histopathologic detections of the colon tumour parts showed significant variability in the colon tissue with less mucosal damage in the MF-treated rats (30 or 60 mg/kg MF) than in the rats received only AOM (Fig. 4).

#### Immunohistochemistry of colon tissues

**Figure 5:**
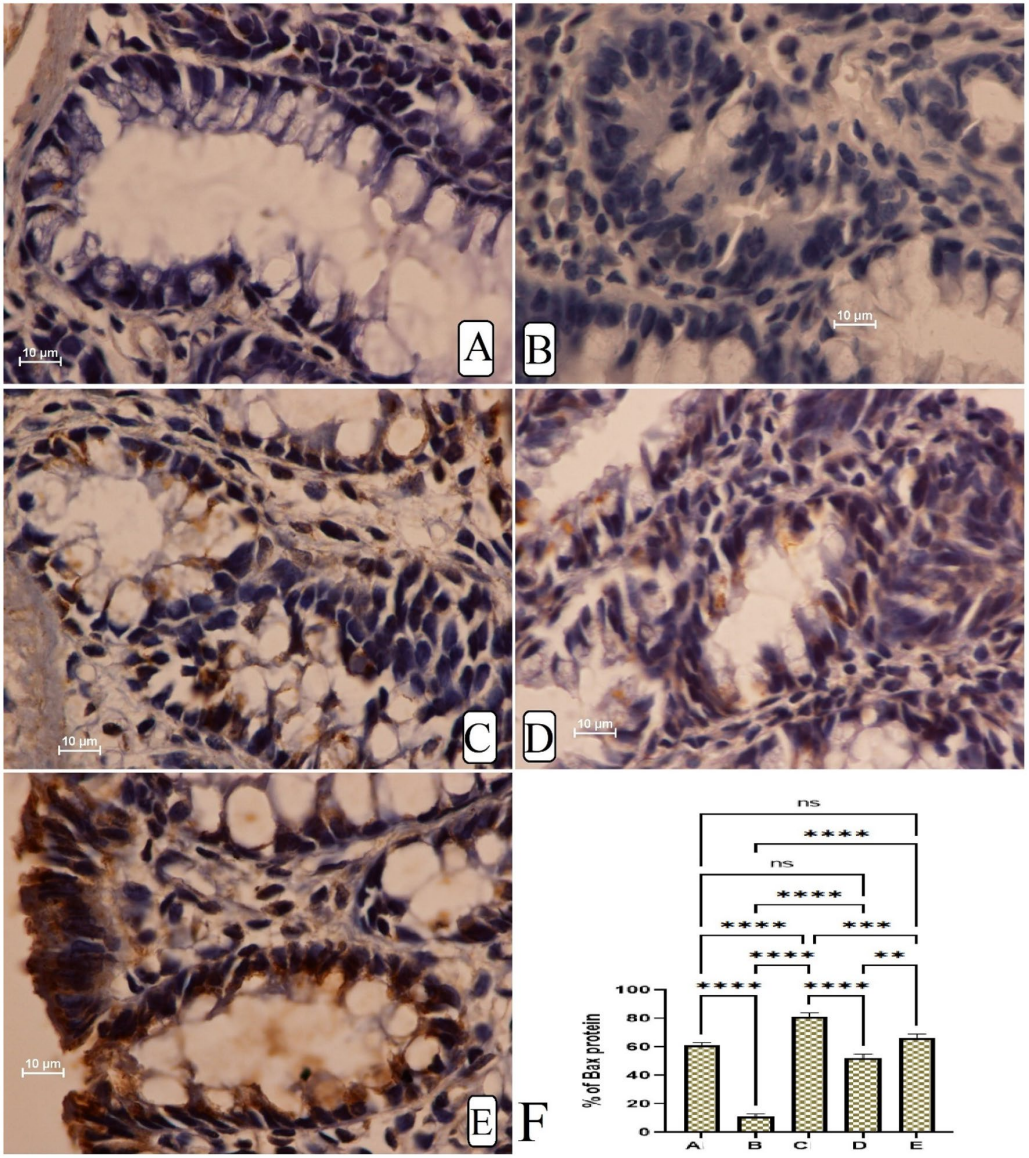
The immunohistochemical appearance of Bax expression in different colon tissues. (**A**) Normal negative rats; (**B**) cancer rats had reduced Bax proteins and severe mucosal injury; (**C**) reference rats (35 mg/kg 5-FU) had the highest Bax protein expression; (**D**, **E**) MF-treated rats (30 and 60 mg/kg MF) showed higher Bax proteins (intense brown colour) with less mucosal damage (magnification, ×100).

**Figure 6:**
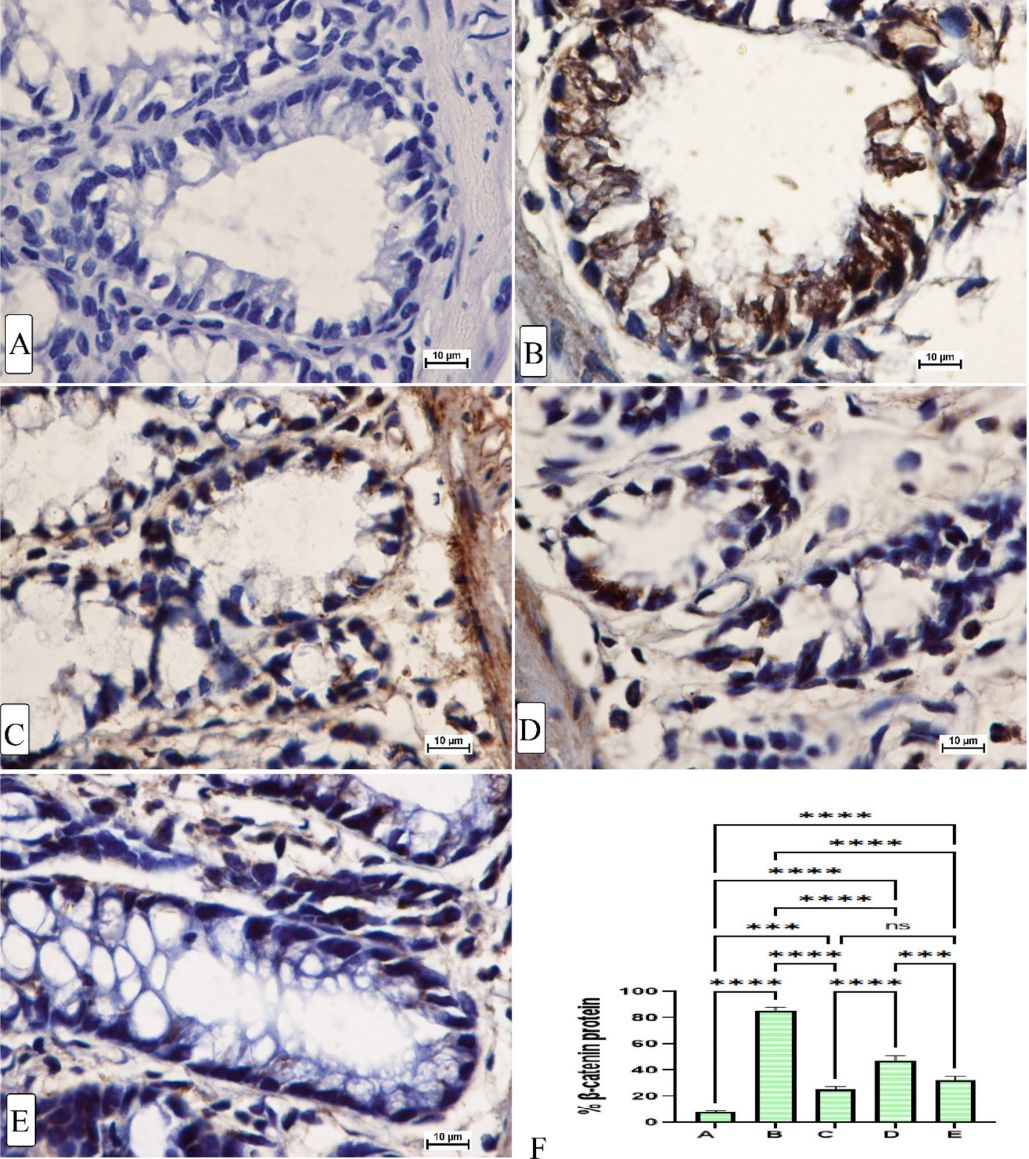
The immunohistochemical appearance of β-catenin protein expression (**A**) normal negative rats; (**B**) cancer rats had elevated β-catenin proteins and severe mucosal injury; (**C**) reference rats (35 mg/kg 5-FU) had the lowest β-catenin protein expression; (**D**, **E**) MF-treated rats (30 and 60 mg/kg MF) showed lower β-catenin proteins (intense brown colour) with less mucosal damage (magnification, ×100).

The current results showed that rats ingested only AOM had significantly lower Bax protein expression (pro-apoptotic factor), thereby facilitating the spreading of tumours across colon tissues and the formation of numerous lesions in mucosal and submucosal layers. MF treatment was found very efficient in the up-regulation of Bax proteins represented by a deep brown colour (Fig. 5A–F). The β-catenin staining intensity was significantly up-regulated in colon tissues of cancer control rats, indicating reduced apoptotic action which will aids in further cell proliferation. AOM treatment lead to different expression of β-catenin proteins in the colon tissue with higher values for cancer control rats than that of MF-treated rats (Fig. 6A–F).

#### Mangiferin effects on enzymatic and non-enzymatic

**Figure 7:**
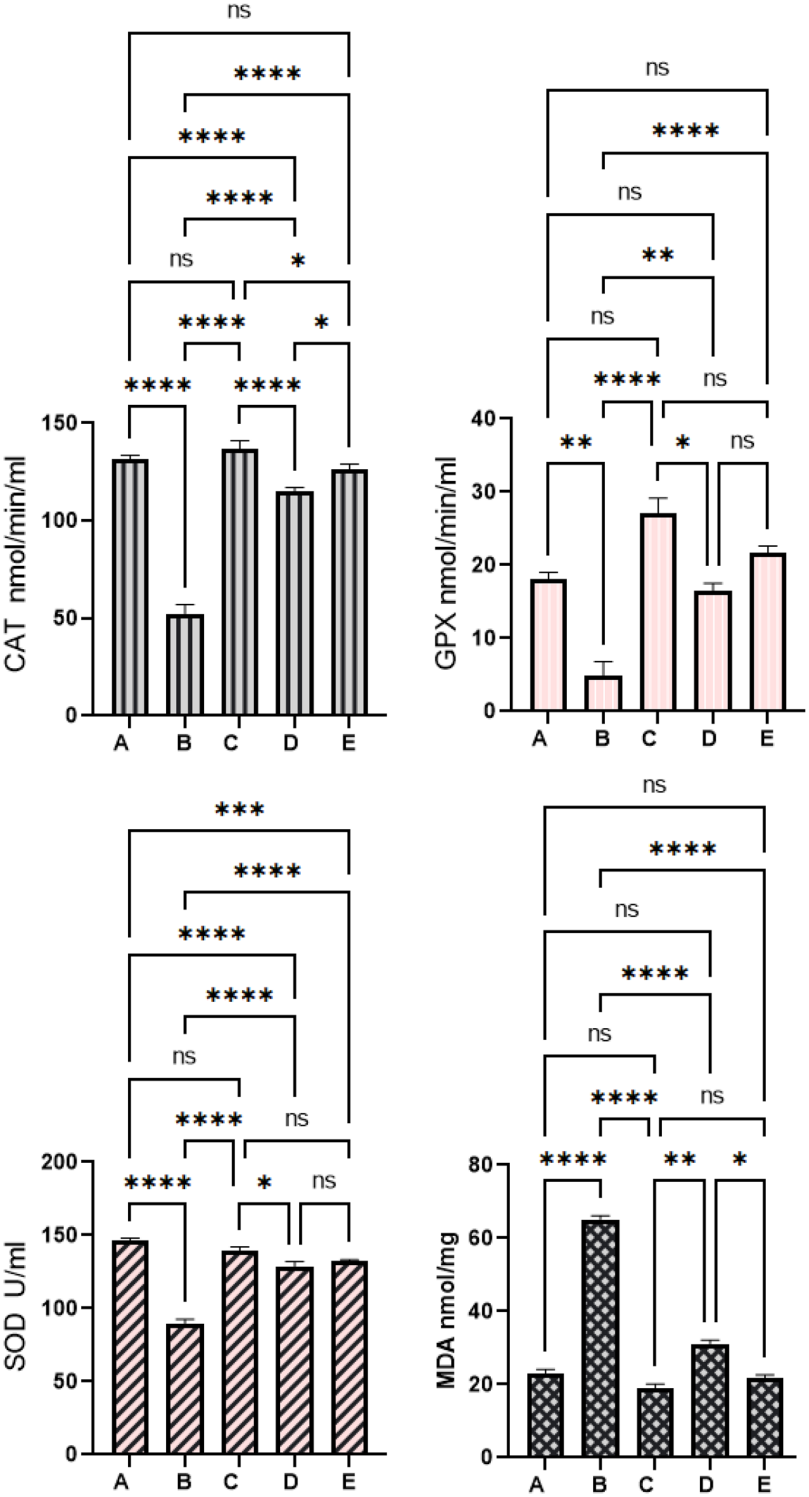
Mangiferin effects on antioxidant parameters. (**A**) Normal negative rats; (**B**) cancer rats treated only with AOM; (**C**) reference rats received 35 mg/kg of 5-FU; (**D**, **E**) rats received 30 and 60 mg/kg MF.

Results of colonic tissue homogenates revealed significant differences in the antioxidant (SOD, CAT, GPx) and MDA contents (Fig. 7). Compared to the normal control rats (A), the antioxidant enzymes were fewer and MDA contents were high in AOM-ingested rats (B). In this context, the antioxidant enzymes were significantly higher and the lipid peroxidation was notably lower in rats received 5-FU (C) or 30 and 60 mg/kg (D and E), respectively. Moreover, rat supplementation with 60 mg/kg MF exposed to AOM significantly up-regulated SOD and down-regulated MDA concentrations to a point that were almost same as the values of 5-FU-treated rats (C).

#### Mangiferin effects on inflammatory cytokines

**Figure 8:**
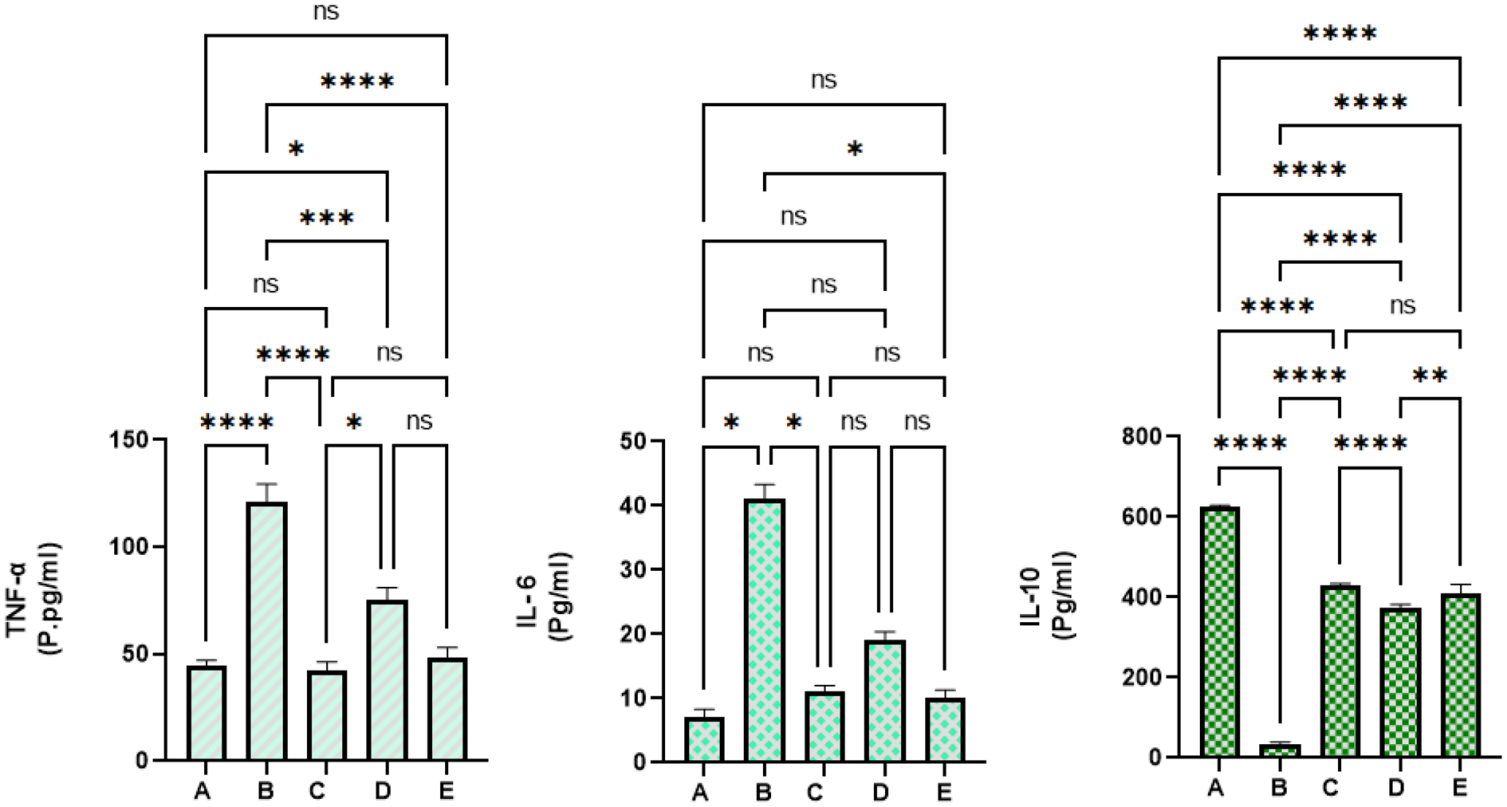
Mangiferin effects on inflammatory cytokines. (**A**) Normal negative rats; (**B**) cancer rats treated only with AOM; (**C**) reference rats received 35 mg/kg of 5-FU; (**D**, **E**) rats received 30 and 60 mg/kg MF.

The current data revealed significant variables in the concentrations of inflammatory cytokines in colon homogenates from experimental rats. Normal control rats showed significantly the lowest values of TNF-α and IL-6 and the highest level of IL-10 compared to all experimental rats. Cancer controls (B) receiving only AOM showed statistically the highest number of pro-inflammatory cytokines (TNF-α and IL-6) and lowest anti-inflammatory cytokines (IL-10) in their tissue homogenates. Mangiferin treatment lead to positive augmentation of inflammatory status in colonic homogenates. Rats fed on a diet supplemented with 30 and 60 mg/kg had significantly higher anti-inflammatory cytokines and lower inflammatory cytokines with enormous statistical variance compared to cancer controls (p > 0.0001) as shown in Fig. 8A–E.

#### Mangiferin effects on serum biochemical parameters

Biochemical results from normal control rats (A) were found within normal range for all estimated parameters. Cancer control rats (B) showed significantly lowest plasma proteins with 76.66 ± 2.9 and 68 ± 3.5 g/L concentrations for total protein and albumin, respectively. Moreover, plasma enzymes (AST and ALT) and kidney tests (urea and creatinine) were notably higher than in the plasma samples obtained from treated rats. Rats treated with reference drug (C) showed good amount of plasma proteins and plasma enzymes in their plasma with normal values (6.71 ± 0.1 and 48.66 ± 2.8 mmol/L) of urea and creatinine, respectively. Mangiferin-treatment (D and E) in AOM-induced foci was associated with significantly higher total proteins and albumin concentrations than in the plasma of cancer control rats. Plasma proteins (ALT, AST, GGT) retained in mangiferin-treated rats and the kidney function tests (urea and creatinine) were significantly down-regulated compared to detected values in the plasma of cancer controls (Table 2).

**Table 2:** Effect of mangiferin on biochemical parameters in AOM-induced colonic ACF in rats.

Groups	Total protein (g/L)	Albumin (g/L)	AST IU/L	ALT (IU/L)	GGT (IU/L)	Urea (mmol/L)	Creatinine (μmol/L)
A	76.66 ± 2.9^b^	19.66 ± 1.9^a^	223.83 ± 2.0^b^	72.5 ± 2.6^b^	6.56 ± 0.4^a^	6.76 ± 0.05^c^	53.66 ± 3.8^b^
B	68 ± 3.5^d^	10.66 ± 1.2^c^	258.33 ± 5.0^a^	88.83 ± 3.1^a^	4.43 ± 0.3^c^	8.21 ± 0.42^a^	65.66 ± 1.9^a^
C	84.16 ± 2.6^a^	20.66 ± 1.7^a^	179.83 ± 5.2^d^	68.16 ± 2.4^c^	6.83 ± 0.18^a^	6.71 ± 0.1^c^	48.66 ± 2.8^c^
D	72.83 ± 3.0^c^	16.16 ± 1.3^b^	206.66 ± 2.6^c^	68.4 ± 2.8^c^	5.1 ± 0.3^b^	7.28 ± 0.49^b^	52.33 ± 4.5^b^
E	78.33 ± 3.6^b^	18.83 ± 2.1^a^	212.16 ± 6.6^b^	71.0 ± 4^b^	6.0 ± 0.3^a^	6.73 ± 0.5^c^	46.33 ± 4.9^c^

Numbers are presented as means ± S.E.M. Means with shared letters considered non-significant P < 0.05. (A) Negative control; (B) cancer control; (C) positive control (5-FU); (D) 30 mg/kg of mangiferin; (E) 60 mg/kg MF.

## Discussion

Medicinal plants have been utilized as effective therapeutics for many organ dysfunctionalities, however, the first barrier to utilizing such natural products is the absence of proven scientific records on their toxicity and adverse effects. Therefore, our study also included toxicity evaluation of MF in two different doses (250 and 500 mg/kg) to set the acceptable healthy dosage for future investigations. Mangiferin treatment did not produce any toxic signs or behavioural changes in rats even after the experimental periods. The current data backup previously conducted a study that reported non-toxic effects of mangiferin after oral ingestion of 250 and 500 mg/kg in rats. Moreover, there were no notable changes in spontaneous locomotor actions^45^.

Natural products has been validated as anticancer and chemoprotective active ingredients because of their regulatory action on different pathways associated with the development, migration, angiogenesis, invasion, and permanent cell arrest. Fruits and vegetables are rich sources of numerous natural compounds that can exhibit different biological actions including antioxidant, anti-inflammatory, and anti-tumour actions^46^. Mangos and honeybush tea are two rich sources of mangiferin that were thought to have anticancer potentials by modulating regulate risk factors associated with cancer initiation and cancer progression^22^.

The colorectal cancer model has been effectively created by using the optimum tolerable dose 10–15 mg/kg of AOM, while a higher dose of 20 mg/kg AOM will lead to complete mortality of animals after one week of ingestion^47^. Furthermore, studies have shown that different routes of administration can lead to different incidence rates of colorectal cancer with researchers reporting 30%, 80%, and 100% for oral, intramuscular, and subcutaneous administrations, respectively^9^. In this context, the current work used 15 mg/kg AOM subcutaneously in rats to evaluate the chemoprotective potentials of MF in the AOM-induced aberrant crypt foci model.

Diagnosing any adenocarcinoma type is possible when tumors, made of different sizes and glandular formats, pass through the muscular layer of colonic tissue^48^. In the present study, rats that ingested only AOM experienced numerous colonic adenomas and adenocarcinomas with notable organ metastasis represented by ileocecal lymph nodes and cecum. Mf treatment leads to a reduction in the amount and volume of colon adenomas and adenocarcinomas. Moreover, MF-treated rats showed significantly lower total ACF than the cancer control rats. Studies investigating MF-rich plants, such as mango, revealed significant inhibitor potentials of this fruit (0.3%) against AOM-induced adenocarcinoma in mice represented by lower ACF values compared to cancer controls^49,50^.

Mucin production is a well-known histological property in colorectal cancer. Mucin amount can be used to classify adenocarcinoma into mucinous adenocarcinoma (aggregation of the extracellular mucin almost 50% of lesions) and the percentage of ACF production. ACF in colon tissues appear with other microscopic features at specific rates, and the amount can be measured by using different microscopic focus^51^. The current study detected increased mucin content and higher ACF values in a typical epithelium of the colon of cancer control rats compared to 5-FU or MF-treated rats.

The apoptotic proteins are well-known modulatory factors that are heavenly impacts the process of cancer progression or termination. The Bax protein as a well-known pro-apoptotic factor increases membrane permeability of the mitochondria and release of cytochrome c, while the β-catenin (an anti-apoptotic factor that preserves outer membrane integrity of the mitochondria) expression in their colon tissues^52^. The β-catenin staining technique is used to assess the level of β-catenin protein expression, which is considered an important regulatory factor to cellular proliferation and aids in T-suppressor cells (aiding in cellular proliferations).The present study showed reduced Bax protein expression and increased β-catenin protein expression in cancer control rats, indicating a significant imbalance between these two proteins could lead to cellular dysfunctionality and changes in the mitochondrial route of apoptosis^53,54^. The present MF (30 and 60 mg/kg) supplementation caused significant positive modulation of Bax protein and noticeably reduction of the β-catenin protein appearance in rat’s colon, which could be a molecular mechanism behind lower ACF incidence and the chemoprotective action of MF in AOM-pre-treated rats. Accordingly, researchers have shown the anticancer potentials of MF in different in vitro cancer studies against leukemia cells^6^ and other cancer (breast, cervix, and prostate) cells^55^, which were mainly correlated with the MF potentials in the regulation of apoptotic factors and expression of proteins associated with the mitotic action. Furthermore, scientists reported increased regulatory action of MF on the apoptotic process in cardiomycytes, which were significantly increased the caspase-3 and Bax protein and reduced the (anti-apoptotic) Bcl-2 protein expressions^30^.

Oxidative stress is one of the main causes related to the increasing rate of inflammatory responses and it has been correlated with the initiation, development, and prognosis of inflammatory bowel disease (IBD)^56^. IBD is a digestive tract disease that mainly affects the large intestine which could be hereditary or result from non-genetic risk factors such as oxidative stress (key pathophysiological). IBD primarily includes Crohn’s disease and ulcerative colitis, which are similar in terms of origin (immunologic overreaction) and differ in their involvement in the digestive system^57^. Moreover, oxidative stress studies have shown the transformation of sensory cells into neoplastic cells in many IBD cases^58^. Therefore, carcinogenesis in the digestive system (colon) includes a sophisticated process that initiates gradually and instantly along with oxidative stress involvement^59,60^. The present work revealed significant antioxidant potentials of MF represented by up-regulation of SOD and CAT, GPx, and down-regulation of lipid peroxidation (MDA) level in colon tissue homogenates. Similarly, numerous studies have shown the antioxidant potentials of mangiferin in up-regulating antioxidant enzymes as well as suppressing reactive oxygen species and lipid peroxidation levels (MDA) in STZ-induced diabetic^61–63^ and acute kidney injury in mice and rats^64^. Moreover, mangiferin was able to retain antioxidant enzymes and lower septic-related organ damage in lipopolysaccharide (LPS)-induced sepsis^65^.

The reactive oxygen species (ROS) can have serious alteration effect on cellular and molecular process including induction of oxidative regulations of cellular proteins (apoptotic proteins). ROS considered as major modulator of growth factors and an enhancer for gene expression, consequently resulting in sustained proliferation of cancer cells^66^. Scientists have shown that ROS overproduction promotes cell grow by modulating the redox status of transcriptional factors and regulatory proteins involved in cell cycle^67^. Apoptosis is well-documented physiological action related with modulation of tissue homeostasis. Apoptosis can be initiated by intracellular (mitochondrial) or extracellular (death receptor) apoptotic mechanisms. Antioxidants play major role in the redox homeostasis, a major regulatory factor of apoptotic pathways (intrinsic or extrinsic)^68^. ROS can have regulatory effect on various apoptotic proteins (caspases, Bax) or anti-apoptotic protein (β-catenin). Increased ROS production has been associated with elevated expression of anti-apoptotic proteins and apoptotic irregularities, subsequently causing various pathological diseases including cancer^69^. Accordingly, the present MF supplementation caused significant up-regulation of antioxidant enzymes, which one of the molecular regulators of lower anti-apoptotic proteins and reduced ACF values in colon tissues of AOM-pretreated rats.

NF-κB is a key modulator in the initiation and development of immune responses and different inflammatory processes. It also facilitates the activation of pro-inflammatory cytokines, IL-6, TNF-α, and prostaglandins. Previous studies have validated that AOM can effectively stimulate the production of inflammatory cytokines and other inflammatory mediators^8,11^. Moreover, TNF-α along with IL-1β can activate the formation of metalloproteinase enzyme and modulates COX-2 overproduction during early phases of carcinogenesis. Interleukin 6 (pro-inflammatory) can activate the JAK/STAT signalling pathways, preventing apoptosis and, along with TNF-α, facilitating angiogenesis and cancer growth^15^. In the current study, rats received only AOM had significantly increased IL-6 and TNF-α cytokines and notably reduced anti-inflammatory cytokine (IL-10) in their blood. Conversely, MF lowered immune and inflammatory responses indicated by up-modulation of IL-6 and TNF-α and down-augmentation of IL-10 cytokines in rats. The outcome suggests significant inhibitory potentials of MF against oxidative stress consequently lead less formation of inflammatory cytokines and inflammation.

Scientists have declared that high mobility group box 1 (HMGB1) protein can conjugate with toll-like receptors (TLR), stimulating NF-κB inflammatory mechanism and inflammatory cytokine release; hence, initiating the inflammation in LPS-treated mice, and notably, mangiferin reversed this effect and down-regulated inflammatory cytokines^65^. Moreover, the antioxidant effects of mango fruit (a major mangiferin source) have been linked with its anti-inflammatory potentials in AOM-induced foci in rats represented by reduced pro-inflammatory cytokines (interleukin 1-Beta, tumor necrosis factor-alpha, interleukin 6, and prostaglandin E2)^70^. The literature cited above validates mangiferin as a bioactive ingredient against various human diseases including chemoprotection against colorectal cancer.

## Conclusion

The present research, based on literature search, is considered the first data on the chemoprotective effects of MF in AOM-induced foci in rats. The current results demonstrate notable cytoprotective actions of MF in AOM-induced colon cancer in rats. MF treatment leads to positive regulation of the pro-apoptotic (Bax) and anti-apoptotic (β-catenin) proteins, increased antioxidant enzymes, lowered pro-inflammatory cytokines, and retained liver and kidney function parameters within the normal range. These bioactivities could be its underline mechanism of chemoprotective potentials. Accordingly, the outcomes present MF as a bioactive ingredient that may serve as viable new source for pharmaceuticals against oxidative stress-mediated disorders including colorectal cancers. The present study faced many limitations including poor facility, lack of specialized instruments, small animal house, and availability of chemical reagents.

## Data Availability

Data details can be provided by corresponding author on request.

## Abbreviations

ALT: Alanine transferase
AOM: Azoxymethane
Bax: Bcl-2-associated X protein
β-catenin: Beta-catenin
CAT: Catalase
FU: Fluorouracil
GPx: Glutathione peroxidase
H and E: Haematoxylin and eosin
IBD: Irritable bowel disease
IL-10: Interleukine-10
IL-6: Interleukine-6
JAK/STAT: Janus kinase/signal transducers and activators of transcription
LDH: Lactate dehydrogenase
LNCaP: Lymph node androgen-dependent human prostatic carcinoma cell
MDA: Malondialdehyde
NF-κB: Nuclear factor kappa-light-chain-enhancer of activated B cells
OECD: Organization for Economic Co-operation and Development
SOD: Superoxide dismutase
TNF-α: Tumour necrosis factor α

## References

[1] Miller KD, Nogueira L, Devasia T, Mariotto AB, Yabroff KR, Jemal A, Kramer J, Siegel RL (2022). Cancer treatment and survivorship statistics, 2022. CA Cancer J. Clin..

[2] Siegel RL, Miller KD, Fuchs HE, Jemal A (2022). Cancer statistics, 2022. CA Cancer J. Clin..

[3] Bardelčíková A, Šoltys J, Mojžiš J (2023). Oxidative stress, inflammation and colorectal cancer: An overview. Antioxidants.

[4] Brianna, Lee SH (2023). Chemotherapy: How to reduce its adverse effects while maintaining the potency?. Med. Oncol..

[5] Wu, I.-T., Kuo, C.-Y., Su, C.-H., Lan, Y.-H. & Hung, C.-C. Pinostrobin and tectochrysin conquer multidrug-resistant cancer cells via inhibiting P-glycoprotein ATPase. *Pharmaceuticals* (2023). 10.3390/ph16020205 PMC9963356 37259354

[6] Norkaew C, Roytrakul S, Charoenlappanit S, Thaisakun S, Tanyong D (2023). Pinostrobin induces acute leukemia cell apoptosis via the regulation of miR-410–5p and SFRP5. Life Sci..

[7] Sayre CL, Alrushaid S, Martinez SE, Anderson HD, Davies NM (2015). Pre-clinical pharmacokinetic and pharmacodynamic characterization of selected chiral flavonoids: Pinocembrin and pinostrobin. J. Pharm. Pharmaceut. Sci..

[8] Jabbar AA, Ibrahim IAA, Abdullah FO, Aziz KF, Alzahrani AR, Abdulla MA (2023). Chemopreventive effects of *Onosma mutabilis* against azoxymethane-induced colon cancer in rats via amendment of Bax/Bcl-2 and NF-κB signaling pathways. Curr. Issues Mol. Biol..

[9] De-Souza ASC, Costa-Casagrande TA (2018). Animal models for colorectal cancer. ABCD Arq. Bras. Cirurgia Digest. (São Paulo).

[10] Uyar A, Doğan A, Yaman T, Keleş ÖF, Yener Z, Çelik İ, Alkan EE (2022). The protective role of *Urtica dioica* seed extract against azoxymethane-induced colon carcinogenesis in rats. Nutr. Cancer.

[11] Ali Abed Wahab B (2023). Pinostrobin attenuates azoxymethane-induced colorectal cytotoxicity in rats through augmentation of apoptotic Bax/Bcl-2 proteins and antioxidants. SAGE Open Med..

[12] Jabbar, A.A., Abdullah, F.O., Abdoulrahman, K., Galali, Y., Ibrahim, I.A., Alzahrani, A.R. & Hassan, R.R. Gastroprotective, biochemical, and acute toxicity effects of *Papaver decaisnei* against ethanol-induced gastric ulcers in rats. *Processes* (2022).

[13] Jabbar AA, Abdullah FO, Hassan AO, Galali Y, Hassan RR, Rashid EQ, Salih MI, Aziz KF (2022). Ethnobotanical, phytochemistry, and pharmacological activity of Onosma (Boraginaceae): An updated review. Molecules.

[14] Jabbar, A.A.J., Alamri, Z.Z., Abdulla, M.A., AlRashdi, A.S., Najmaldin, S.K. & Zainel, M.A. Sinapic acid attenuate liver injury by modulating antioxidant activity and inflammatory cytokines in thioacetamide-induced liver cirrhosis in rats. *Biomedicines* (2023). 10.3390/biomedicines11051447 PMC10216417 37239118

[15] Esmeeta A, Adhikary S, Dharshnaa V, Swarnamughi P, Ummul Maqsummiya Z, Banerjee A, Pathak S, Duttaroy AK (2022). Plant-derived bioactive compounds in colon cancer treatment: An updated review. Biomed. Pharmacother..

[16] Nardella F, Dobrescu I, Hassan H, Rodrigues F, Thiberge S, Mancio-Silva L, Tafit A, Jallet C, Cadet-Daniel V, Goussin S (2023). Hemisynthetic alkaloids derived from trilobine are antimalarials with sustained activity in multidrug-resistant *Plasmodium falciparum*. iScience.

[17] Albeshri, A., Baeshen, N.A., Bouback, T.A. & Aljaddawi, A.A. A review of *Rhazya stricta* decne phytochemistry, bioactivities, pharmacological activities, toxicity, and folkloric medicinal uses. *Plants *(2021). 10.3390/plants10112508 PMC8619226 34834871

[18] Jantan I, Arshad L, Septama AW, Haque MA, Mohamed-Hussein Z, Govender NT (2023). Antiviral effects of phytochemicals against severe acute respiratory syndrome coronavirus 2 and their mechanisms of action: A review. Phytother. Res..

[19] Rabidas SS, Prakash C, Tyagi J, Suryavanshi J, Kumar P, Bhattacharya J, Sharma D (2023). A comprehensive review on anti-inflammatory response of flavonoids in experimentally-induced epileptic seizures. Brain Sci..

[20] Wahab BAA (2023). Phytochemistry, antioxidant, anticancer, and acute toxicity of traditional medicinal food Biarum bovei (Kardeh). BMC Complement. Med. Ther..

[21] Jabbar AA (2023). Phytochemical profile, antioxidant, enzyme inhibitory and acute toxicity activity of *Astragalus bruguieri*. Baghdad. Sci. J..

[22] Gold-Smith F, Fernandez A, Bishop K (2016). Mangiferin and cancer: Mechanisms of action. Nutrients.

[23] Morozkina SN, Nhung Vu TH, Generalova YE, Snetkov PP, Uspenskaya MV (2021). Mangiferin as new potential anti-cancer agent and mangiferin-integrated polymer systems—A novel research direction. Biomolecules.

[24] Tohamy H-AS, El-Sakhawy M, El-Masry HM, Saleh IA, AbdelMohsen MM (2022). Preparation of hydroxyethyl cellulose/mangiferin edible films and their antimicrobial properties. BMC Chem..

[25] Shen J, Lu R, Cai Q, Fan L, Yan W, Zhu Z, Yang L, Cao Y (2021). Mangiferin enhances the antifungal activities of caspofungin by destroying polyamine accumulation. Virulence.

[26] Wang M, Liang Y, Chen K, Wang M, Long X, Liu H, Sun Y, He B (2022). The management of diabetes mellitus by mangiferin: Advances and prospects. Nanoscale.

[27] Chang B, Jiang H, Wei Y, Gong Q, Yu D, Dong Z, Luo J, Gao Y, Yao Q (2022). Mangiferin: Analgesic properties in neuropathic pain, molecular docking and meta-analysis. Phytomed. Plus.

[28] Saha S, Sadhukhan P, Sil PC (2016). Mangiferin: A xanthonoid with multipotent anti-inflammatory potential. Biofactors.

[29] Chen L, Li S, Zhu J, You A, Huang X, Yi X, Xue M (2021). Mangiferin prevents myocardial infarction-induced apoptosis and heart failure in mice by activating the Sirt1/FoxO3a pathway. J. Cell. Mol. Med..

[30] Jiang T, Han F, Gao G, Liu M (2020). Mangiferin exert cardioprotective and anti-apoptotic effects in heart failure induced rats. Life Sci..

[31] Khurana RK, Gaspar BL, Welsby G, Katare OP, Singh KK, Singh B (2018). Improving the biopharmaceutical attributes of mangiferin using vitamin E-TPGS co-loaded self-assembled phosholipidic nano-mixed micellar systems. Drug Deliv. Transl. Res..

[32] Ansari Z, Goomer S (2022). Natural gums and carbohydrate-based polymers: Potential encapsulants. Indo Glob. J. Pharmaceut. Sci..

[33] Kaur P, Gupta RC, Dey A, Malik T, Pandey DK (2021). Optimization of harvest and extraction factors by full factorial design for the improved yield of C-glucosyl xanthone mangiferin from *Swertia chirata*. Sci. Rep..

[34] Mei S, Ma H, Chen X (2021). Anticancer and anti-inflammatory properties of mangiferin: A review of its molecular mechanisms. Food Chem. Toxicol..

[35] Gendy AM, El-Gazar AA, Ragab GM, Al-Mokaddem AK, El-Haddad AE, Selim HMRM, Yousef EM, Hamed NO, Ibrahim SSA (2022). Possible implication of Nrf2, PPAR-γ and MAPKs signaling in the protective role of mangiferin against renal ischemia/reperfusion in rats. Pharmaceuticals.

[36] Furman D, Campisi J, Verdin E, Carrera-Bastos P, Targ S, Franceschi C, Ferrucci L, Gilroy DW, Fasano A, Miller GW, Miller AH, Mantovani A, Weyand CM, Barzilai N, Goronzy JJ, Rando TA, Effros RB, Lucia A, Kleinstreuer N, Slavich GM (2019). Chronic inflammation in the etiology of disease across the life span. Nat. Med..

[37] Fantini MC, Guadagni I (2021). From inflammation to colitis-associated colorectal cancer in inflammatory bowel disease: Pathogenesis and impact of current therapies. Digest. Liver Dis..

[38] Sirois, M. *Laboratory an for Laboratory Animal Experiments Imal and Exotic Pet Medicine-e-Book: Principles and Procedures* (2022).

[39] Ab.jabbar A (2021). Onosma mutabilis: Phytochemical composition, antioxidant, cytotoxicity, and acute oral toxicity. Food Sci. Nutr..

[40] Mariod AA, Jabbar AAJ, Alamri ZZ, Rashdi ASA, Abdulla MA (2023). Gastroprotective effects of *Polygonatum odoratum* in rodents by regulation of apoptotic proteins and inflammatory cytokines. Saudi J. Biol. Sci..

[41] Al-Medhtiy MH, Jabbar AA, Shareef SH, Ibrahim IAA, Alzahrani AR, Abdulla MA (2022). Histopathological evaluation of *Annona muricata* in TAA-induced liver injury in rats. Processes.

[42] David SRN, Mohammad MS, Chee LY, Rajabalaya R (2022). Is sunflower cooking oil beneficial for colorectal cancer? In vivo studies on azoxymethane-induced colon cancer in rats. Curr. Nutr. Food Sci..

[43] Al-Henhena N, Khalifa SAM, Ying RPY, Hassandarvish P, Rouhollahi E, Al-Wajeeh NS, Ali HM, Abdulla MA, El-Seedi HR (2015). Chemopreventive effects of *Strobilanthes crispus* leaf extract on azoxymethane-induced aberrant crypt foci in rat colon. Sci. Rep..

[44] Jabbar AAJ (2023). Hepatoprotective effects of *Gynura* procumbens against thioacetamide-induced cirrhosis in rats: Targeting inflammatory and oxidative stress signalling pathways. Heliyon.

[45] Dimitrov M, Nikolova I, Benbasat N, Kitanov G, Danchev N (2011). Acute toxicity, antidepressive and MAO inhibitory activity of mangiferin isolated from *Hypericum aucheri*. Biotechnol. Biotechnol. Equip..

[46] Huang M, Lu J-J, Ding J (2021). Natural products in cancer therapy: Past, present and future. Nat. Prod. Bioprospect..

[47] Chen J, Huang X-F (2009). The signal pathways in azoxymethane-induced colon cancer and preventive implications. Cancer Biol. Ther..

[48] Sharma A, Kumar R, Yadav G, Garg P (2023). Artificial intelligence in intestinal polyp and colorectal cancer prediction. Cancer Lett..

[49] Rajendran P, Rengarajan T, Nandakumar N, Divya H, Nishigaki I (2015). Mangiferin in cancer chemoprevention and treatment: pharmacokinetics and molecular targets. J. Recept. Signal. Transduct..

[50] Rahmani AH (2023). Role of mangiferin in management of cancers through modulation of signal transduction pathways. Biomedicines.

[51] Leopoldo S, Lorena B, Cinzia A, Gabriella DC, Angela Luciana B, Renato C, Antonio M, Carlo S, Cristina P, Stefano C (2008). Two subtypes of mucinous adenocarcinoma of the colorectum: Clinicopathological and genetic features. Ann. Surg. Oncol..

[52] Zhang Y, Li Q, Zhou D, Chen H (2013). Genistein, a soya isoflavone, prevents azoxymethane-induced up-regulation of WNT/β-catenin signalling and reduces colon pre-neoplasia in rats. Br. J. Nutr..

[53] Ozdemir O, Akcakavak G, Tuzcu M (2022). Efecto del extracto alcohólico de Tarantula cubensis y el destilado de *Nerium oleander* sobre los marcadores de proliferación celular en la carcinogénesis de colon. Rev. Cien. Fac. Cien. Vet. Univ. Zulia.

[54] Pu J, Zhou X, Liu J, Hou P, Ji M (2021). Therapeutic potential and deleterious effect of glucocorticoids on azoxymethane/dextran sulfate sodium-induced colorectal cancer in mice. Am. J. Cancer Res..

[55] Núñez Selles AJ, Daglia M, Rastrelli L (2016). The potential role of mangiferin in cancer treatment through its immunomodulatory, anti-angiogenic, apoptopic, and gene regulatory effects. BioFactors.

[56] Tian T, Wang Z, Zhang J (2017). Pathomechanisms of oxidative stress in inflammatory bowel disease and potential antioxidant therapies. Oxid. Med. Cell. Longev..

[57] Xu S, Li X, Zhang S, Qi C, Zhang Z, Ma R, Xiang L, Chen L, Zhu Y, Tang C (2023). Oxidative stress gene expression, DNA methylation, and gut microbiota interaction trigger Crohn’s disease: A multi-omics Mendelian randomization study. BMC Med..

[58] De Stefano L, Pallavicini FB, Mauric E, Piccin V, Vismara EM, Montecucco C, Bugatti S (2023). Tumor necrosis factor-α inhibitor-related immune disorders. Autoimmun. Rev..

[59] Babu SSN, Singla S, Jena G (2023). Role of combination treatment of aspirin and zinc in DMH-DSS-induced colon inflammation, oxidative stress and tumour progression in male BALB/c mice. Biol. Trace Elem. Res..

[60] Lee I-H, Lee D-Y (2019). FDY003 inhibits colon cancer in a Colo205 xenograft mouse model by decreasing oxidative stress. Pharmacogn. Mag..

[61] Sekar V, Mani S, Malarvizhi R, Barathidasan R, Vasanthi HR (2020). Positive interaction of mangiferin with selected oral hypoglycemic drugs: A therapeutic strategy to alleviate diabetic nephropathy in experimental rats. Mol. Biol. Rep..

[62] Song Y, Liu W, Tang K, Zang J, Li D, Gao H (2020). Mangiferin alleviates renal interstitial fibrosis in streptozotocin-induced diabetic mice through regulating the PTEN/PI3K/Akt signaling pathway. J. Diabetes Res..

[63] Wang X, Gao L, Lin H, Song J, Wang J, Yin Y, Zhao J, Xu X, Li Z, Li L (2018). Mangiferin prevents diabetic nephropathy progression and protects podocyte function via autophagy in diabetic rat glomeruli. Eur. J. Pharmacol..

[64] He L, Peng X, Zhu J, Chen X, Liu H, Tang C, Dong Z, Liu F, Peng Y (2014). Mangiferin attenuate sepsis-induced acute kidney injury via antioxidant and anti-inflammatory effects. Am. J. Nephrol..

[65] Zhang D, Han S, Zhou Y, Qi B, Wang X (2020). Therapeutic effects of mangiferin on sepsis-associated acute lung and kidney injuries via the downregulation of vascular permeability and protection of inflammatory and oxidative damages. Eur. J. Pharmaceut. Sci..

[66] Harris IS, Treloar AE, Inoue S, Sasaki M, Gorrini C, Lee KC, Yung KY, Brenner D, Knobbe-Thomsen CB, Cox MA (2015). Glutathione and thioredoxin antioxidant pathways synergize to drive cancer initiation and progression. Cancer Cell.

[67] Abduljabbar AA, Ismail PA (2019). Investigation of malondialdehyde (MDA), homocysteine (Hcy) and C-reactive protein (CRP) in sera of patients with angina pectoris. Al-Mustansiriyah J. Sci..

[68] Kang Ah K, Piao Jing M, Ryu Seong Y, Hyun Jae Y, Park Eon J, Shilnikova K, Zhen Xuan A, Kang Kyoung H, Koh Sang Y, Jeong Joo Y, Hyun Won J (2017). Luteolin induces apoptotic cell death via antioxidant activity in human colon cancer cells. Int. J. Oncol..

[69] Osman Mahmud, S. *et al*. Green synthesis of silver nanoparticles from aqueous extract of *Tinospora crispa* stems accelerate wound healing in rats. *Int. J. Low Extrem. Wounds* 15347346221133628. 10.1177/15347346221133627 (2022). 10.1177/15347346221133627 36325727

[70] Corrales-Bernal A, Jaramillo G, Rodríguez B, Kazuz EY, Maldonado-Celis ME (2016). Mango (*Mangifera indica* cv. *Azúcar*) antiinflammatory and chemopreventive role during colorectal carcinogenesis. Emirates J. Food Agric..

